# Evaluation of Diffusion-Weighted MRI and FDG-PET/CT to Assess Response to AdCD40L treatment in Metastatic Melanoma Patients

**DOI:** 10.1038/s41598-019-54438-x

**Published:** 2019-12-02

**Authors:** Aglaia Schiza, Sandra Irenaeus, Francisco Ortiz-Nieto, Angelica Loskog, Thomas Tötterman, Anders Sundin, Gustav J. Ullenhag, Håkan Ahlström

**Affiliations:** 1 Department of Immunology, Genetics and Pathology, Science for Life Laboratory, Uppsala University, Dag Hammarskjoldsvag 20, 751 85 Uppsala, Sweden; 2 Department of Oncology, Uppsala University Hospital, 751 85 Uppsala, Sweden; 3 Department of Surgical Sciences, Uppsala University, 751 85 Uppsala, Sweden; 4 Department of Radiology, Uppsala University Hospital, 751 85 Uppsala, Sweden; 5 Antaros Medical AB, Mölndal, Sweden; 6 Lokon Pharma AB, Uppsala Science Park, 751 83 Uppsala, Sweden

**Keywords:** Cancer imaging, Cancer immunotherapy, Melanoma

## Abstract

The purpose was to evaluate the potential of diffusion-weighted-magnetic resonance imaging (DW-MRI) and ^18^F-fludeoxy-glucose-positron emission tomography integrated with CT (FDG-PET/CT) for prediction of overall survival (OS) following AdCD40L-immunotherapy in patients with metastatic malignant melanoma (MMM). Twenty-four patients with refractory MMM were treated with immunostimulatory AdCD40L gene therapy in a phase I/IIa study. Pre-therapeutic DW-MRI and FDG-PET/CT were performed and then repeated at 5 and 9 weeks post-treatment. Evaluation was conducted according to RECIST 1.1 and EORTC criteria. Apparent diffusion coefficient (ADC), true diffusion coefficient (D), maximum standardized uptake value (SUV_max_) were measured in the injected lesions. Fold changes (*F*) in ADC (*F ADC*), D (*F D*), SUV_max_ (*F SUV*_*max*_) were statistically assessed. *F D* ≥ 1 and F *ADC* ≥ 1 were associated with better OS in scans at week 5 and 9 respectively. F SUVmax was not correlated to OS. F *ADC* ≥ 1 in both post-treatment scans and *F D* ≥ 1 at week 5 were related to a significant decrease of size of the injected lesions. These results suggest that in patients with MMM treated with AdCD40l, functional parameters of DW-MRI are better early predictors of OS than the established metabolic and morphologic criteria for FDG-PET/CT and MRI, respectively.

## Introduction

Melanoma is a malignant disease that arises from melanocytes and has a steadily increased incidence over the last decades^1,2^. This rising trend is expected to continue in the future^3^.

Historically, the prognosis of patients with disseminated melanoma has been poor, with an approximately 10% 5-year survival^4,5^. During the last years, the prognosis has been substantially improved due to gradual understanding of the biology of melanoma and the access to new therapeutic options as targeted therapies and immunotherapy^6–8^. Advances related to immunotherapy are reflected on the current 5-year survival rate at approximately 35%^9^. Immunotherapy has posed challenges to clinicians due to the different mechanisms of action compared to chemotherapy, which can lead to atypical patterns of response and immune-related adverse events.

Despite of the advances in treatment options there is still a need for effective drugs with less side effects^10^ and for methods that enable early evaluation of treatment response to swiftly identify non-responders^11^. Immunotherapy has been established in recent years and has evolved to a cornerstone in the treatment of many cancers^12,13^. Response to immunotherapy is still mainly based on assessment of computed tomography (CT) and magnetic resonance imaging (MRI) according to RECIST 1.1^14^ although benefits of immunotherapy may not require significant reduction in tumor size^15^ and therefore functional methods have been applied. In fact, an atypical pattern of response, pseudoprogression, has been observed in about 6–10% of patients undergoing immunotherapy, in whom an initial tumor increase or appearance of new lesions is followed by subsequent decrease of tumor burden^16,17^.

CT remains the basic method for therapy monitoring of MM^15^ although MRI and ^18^F-fludeoxy-glucose (FDG)-positron emission tomography (PET) integrated with CT (PET/CT) are increasingly employed. FDG-PET/CT is helpful for therapy monitoring of various cancers^18–20^ but is expensive and shortly following radiotherapy and surgery it may be difficult to differ tumor related FDG-uptake from that induced by inflammation^15^. In 1999, the European Organization for Research and Treatment of Cancer (EORTC) proposed the first PET-based response criteria to assess therapy response in solid tumors^21,22^. The PET Response Criteria in Solid Tumors (PERCIST) were launched in 2009. With the PERCIST, SUV (normalized by body-weight) was replaced by SUV lean (normalized by lean body mass), and measurements of up to five target lesions were introduced^23,24^. The RECIST 1.1 have been adapted for assessment of response to immunotherapies. Whereas new lesions in RECIST 1.1 always result in progressive disease (PD) this is not the case in iRECIST or irRECIST; iRECIST require verification of unconfirmed progressive disease by a follow-up scan^25^. The most recent concept is iPERCIST that also take into account the possible avid-FDG lymphocytic tumoral infiltration during treatment^26^.

Diffusion-weighted - magnetic resonance imaging (DW-MRI) is routinely included in MRI examination protocols for tumor diagnosis, to differ benign from malignant lesions, to detect recurrent disease, and to assess treatment response^27,28^. DW-MRI is based on the random movement of water molecules. This random movement is restricted in hypercellular tissues such as in tumors^29^, resulting in low values of the apparent diffusion coefficient (ADC). ADC, a measure of the magnitude of diffusion of water molecules within tissue, is calculated by using the mono-exponential decay of signals for b-values over 100 s/mm^2^^30^. However, the DW imaging (DWI) signal is influenced aside from molecular diffusion also by pseudorandom motion in the capillary network (perfusion). The intra voxel incoherent motion (IVIM) model is a refined analysis technique to separate perfusion- and diffusion-related effects by assuming biexponential behavior of signal decay^30–32^. IVIM-derived parameters include the true diffusion coefficient (D) and perfusion-related coefficient (D*) and perfusion fraction (f). Changes in IVIM parameters may be useful for early tumor response assessment as observed in animal studies^33^. Due to the major advances in the field of DW-MRI and at the same time lack of repeatability literature, the Quantitative Imaging Biomarkers Alliance (QIBA) has documented DWI profiles based on review of many published studies. The fact that varying DW-MRI protocols are applied in different organs is an issue. According to QIBA, a substantial difference in the measurements of ADC must be observed to ensure that a significant change has occurred. However, Paudyal *et al*. have reported preliminary findings for repeated IVIM- measurements where smaller differences were considered significant^34^.

Although FDG-PET/CT is established in the clinical praxis to assess immunotherapy responses in Hodgkin lymphoma and Head&Neck and lung cancer, almost having replaced CT^35^ the role of DW-MRI in this regard is not yet equally established.

In a phase I/IIa study, adenovirus carrying the transgene for human CD40 ligand (AdCD40L) was repeatedly administered intratumorally in the same preselected lesion by ultrasound-guidance injections in patients with disseminated MM having previously received established treatments. AdCD40L induced desirable systemic immune effects, which correlated to prolonged survival^36–38^.

The purpose of the current study was to evaluate the potential of DW-MRI and FDG-PET/CT for prediction of OS following AdCD40L immunotherapy in patients with MMM. We hypothesized that the quantitative values of DW-MRI and FDG-PET/CT could predict tumor response before tumor shrinkage occurs. We furthermore hypothesized that DW-MRI measurements should be at least as reliable as PET-measurements in our cohorts.

## Results

All patients who underwent at least one post-treatment examination (n = 21), the evaluable patients, were analyzed (Table 1). Patients #11, #22 and #23 suffered from rapid PD and did not complete the treatment scheme. Patients #1, #6, #9 and #21 did not undergo Scans 2 (w 9) due to deterioration of their general condition. Patient #2 did not undergo MRI-scan 2 since she received a non-MRI-compatible esophageal stent. Patient #3 could not undergo PET-scan 2 due to pneumonia. No DWI-data could be recovered on patients # 20 and #24. As a result, data from all examinations were available only in thirteen patients (n = 13).

**Table 1:** Radiological evaluation by MRI and FDG-PET/CT in patients with disseminated malignant melanoma treated with AdCD40L.

MRI evaluation (RECIST 1.1)			PET evaluation (EORTC criteria)	
Patient no	Week 5	Week 9	Week 5	Week 9
	*Scan 1*	*Scan 2*	*Scan 1*	*Scan 2*
**Without cyclophosphamide**				
1	PD	—	SMD	—
2	PD	—	PMR	PMR
3	SD	PD	PMR	—
4	SD	SD	SMD	SMD
5	SD	SD	SMD	SMD
6	PD	—	SMD	—
**With cyclophosphamide**				
7	SD	SD	SMD	SMD
8	SD	SD	PMR	SMD
9	PD	—	PMR	—
10	SD	SD	SMD	SMD
11	—	—	—	—
12	PD	PD	SMD	PMR
13	PD	PD	PMD	SMD
14*	SD	PD	SMD	SMD
15	PD	PD	SMD	SMD
**With cyclophosphamide and irradiation**				
16	PD	PD	PMD	PMD
17	SD	PD	SMD	PMR
18	SD	SD	PMR	PMR
19	SD	SD	PMD	PMD
20	SD	SD	PMR	PMR
21	PD	—	SMD	—
22	—	—	—	—
23	—	—	—	—
24*	PD	PD	PMD	PMD

Abrreviations: – = not done; SD = stable disease; PD = progressive disease; PMD = progressive metabolic disease; PMR = partial metabolic response; SMD = stable metabolic disease.

Treatment response assessments by RECIST 1.1. and EORTC criteria. *Due to technical issues the DWI-data were not available.

The DWI and PET parameters in the injected metastases were investigated in the three cohorts using a non-parametric Kruskal-Wallis test and were all found similar between cohorts (Supplemental Material, Table 1s).

### Associations between fold changes of DWI/PET parameters and OS

#### All evaluable patients (n = 21)

**Figure 1:**
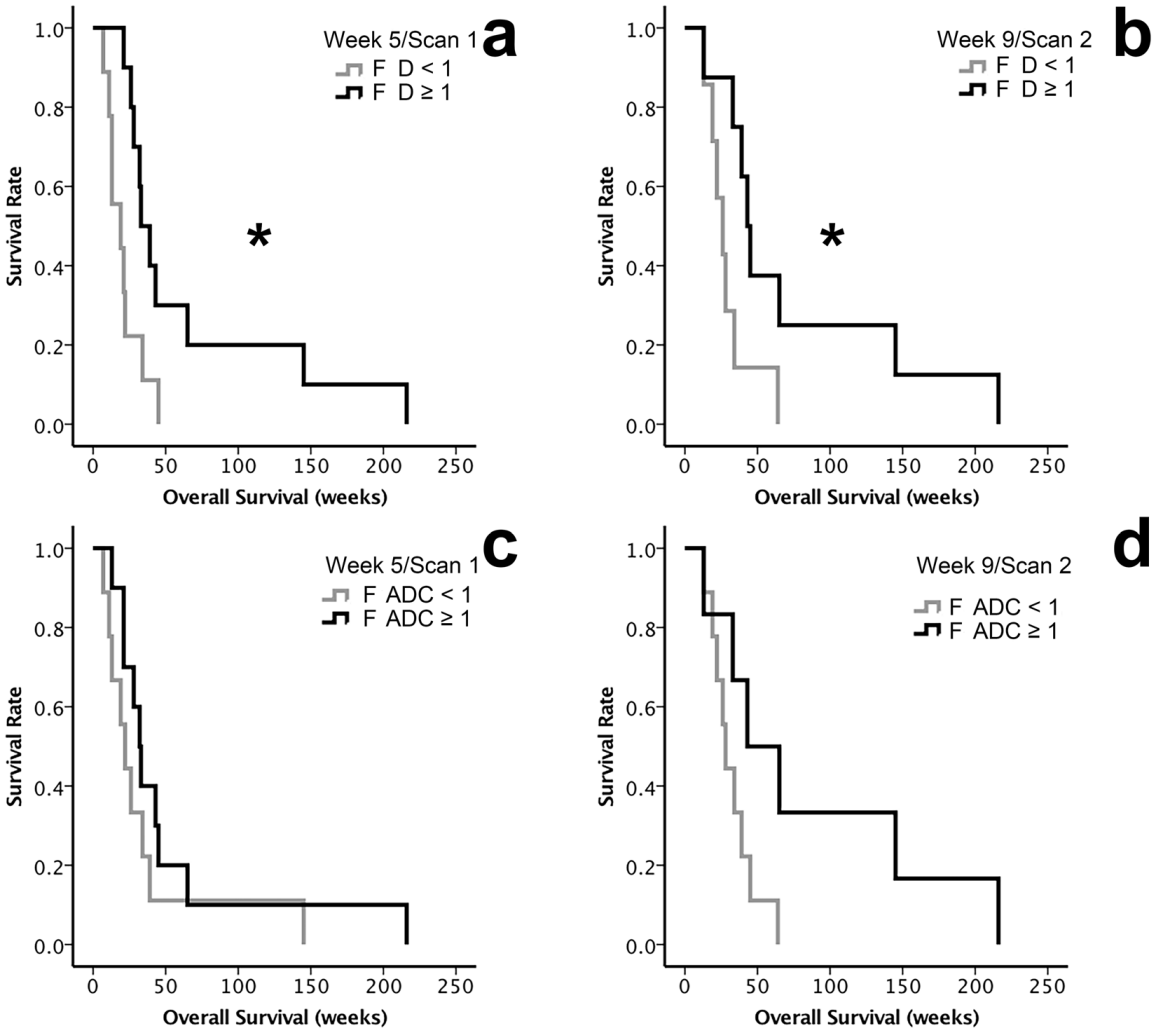
Overall survival (OS) of patients with metastatic melanoma treated with AdCD40L gene therapy assessed by fold change (*F*) *F ADC*, *F D* and *F SUV*_*max*_ of the injected metastases. The analyses were conducted with inclusion of all patients who underwent at least one post-treatment examination (FDG-PET/CT or DW-MRI, n = 21). The Kaplan-Meier method was used followed by log-rank tests. (**a**) The median OS of the patients with *F D* ≥ 1 (black line) compared to the median OS of the patients with *F D* < 1 (grey line) in Scan 1 at week 5 (p = 0.013). (**b**) The median OS of the patients with *F D* ≥ 1 (black line) compared to the median OS of the patients with *F D* < 1 (grey line) in Scan 2 at week 9 (p = 0.032).(**c,d**) The median OS between patients with *F ADC* ≥ 1 (black lines) and compared with those with *F ADC* < 1 (grey lines) in Scan 1 at week 5 (c; p = 0.251) and in Scan 2 at week 9 (d; p = 0.057).

*F D* and *OS*. The OS of patients with *F D* ≥ 1 and *F D* < 1 of the injected metastases in Scan1 (at week 5) and Scan2 (at week 9) are shown in Fig. 1a,b (Table 2). In Scan1 the median OS of patients with *F D* ≥ 1 (n = 10) was 33 ± 5.5 weeks and with *F D* < 1 (n = 9) was 19 ± 8.9 weeks (p = 0.013). In Scan2 the median OS was 43 ± 4.2 weeks when *F D* ≥ 1 (n = 6) and 26 ± 5.2 weeks when *F D* < 1 (n = 9; p = 0.032).

**Table 2:** Associations between fold changes of DWI-parameter D and ADC of the injected metastases and OS, in all evaluable patients (n = 21) and in patients who underwent all the evaluation scans (n = 13).

n	Parameters	Scan 1 (weeks)	Scan 2 (weeks)
13	*F D < 1*	22 ± 3.3	22 ± 4.3
	*F D* ≥ 1	39 ± 7.1	45 ± 2.6
	*F ADC < 1*	26 ± 5.2	26 ± 4.2
	*F ADC* ≥ 1	43 ± 7.3	65 ± 24.1
21	*F D < 1*	19 ± 8.9	22 ± 4.3
	*F D* ≥ 1	33 ± 5.5	45 ± 2.6
	*F ADC < 1*	22 ± 4.5	28 ± 3
	*F ADC* ≥ 1	32 ± 4	43 ± 20

*F ADC* and *OS*. A similar pattern was observed for OS regarding *F ADC* of the injected metastasis in Scan2 (p = 0.251 at week 5, Fig. 1c and p = 0.057 at week 9, Fig. 1d).

*F SUV*_*max*_, F *f* and OS. Similar analyses for *F SUV*_*max*_ and F *f* showed no difference in OS for *F SUV*_*max*_ and *F f* ≥ 1 and < 1 (Supplemental Material, Figs. 1s–4s).

#### Patients who underwent all the evaluation scans (n = 13)

**Figure 2:**
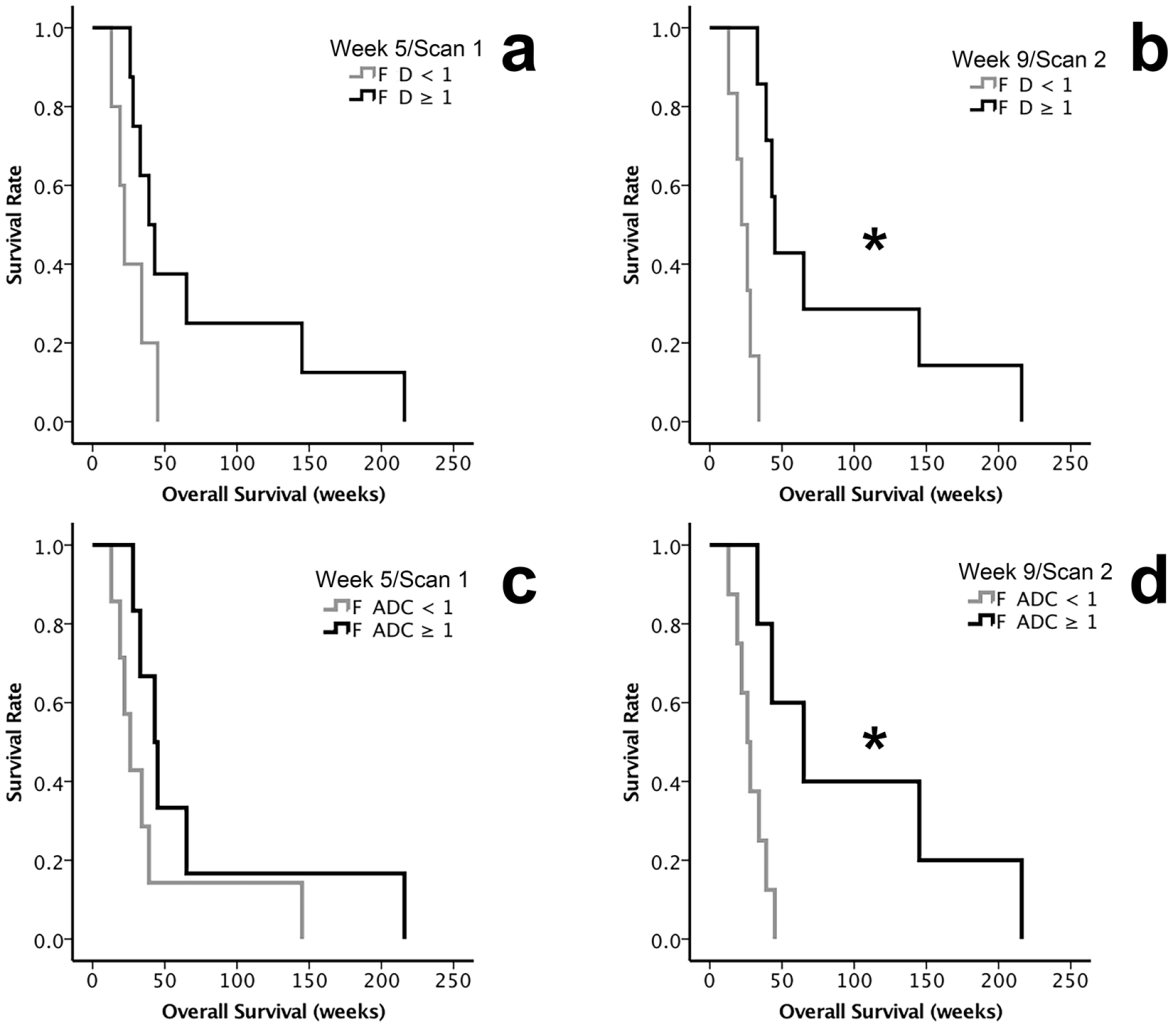
Overall survival (OS) of patients with metastatic melanoma treated with AdCD40L gene therapy assessed by fold change (*F*) *F ADC*, *F D* and *F SUV*_*max*_ of the injected metastases. The analyses were conducted excluding the patients who did not undergo all examinations (n = 13). The Kaplan-Meier method was used followed by log-rank tests. (**a**) The median OS of patients with *F D* ≥ 1 (black lines) compared with those with *F D* < 1 (grey lines) observed in Scan 1 at week 5 (p = 0.075).(**b**) The median OS of the patients with *F D* ≥ 1 (black line) compared with the median OS of the patients with *F D* < 1 (grey line) in Scan 2 at week 9 (p = 0.001).(**c**) Comparison of the median OS in patients with *F ADC* ≥ 1 (black lines) and those with *F ADC* < 1 (grey lines) in Scan 1 at week 5 (p = 0.15).(**d**) Comparison of the median OS of the patients with *F ADC* ≥ 1 (black line) to the median OS of the patients with *F ADC* < 1 (grey line) in Scan 2 at week 9 (p = 0.014). (* indicates p < 0.05).

*F D* and *OS*. The OS of patients with *F D* ≥ 1 and *F D* < 1 of the injected metastases in Scan 1 and 2 are shown in Fig. 2a (p = 0.075) and b (p = 0.001) (Table 2). In Scan1, the median OS of patients with *F D* ≥ 1 (n = 8) was 39 ± 7.1 weeks and with *F D < *1 (n = 5) was 22 ± 3.3 weeks. In Scan 2, the median OS were 45 ± 2.6 weeks when *F D* ≥ 1 (n = 7) and 22 ± 4.3 weeks when *F D* < 1 (n = 6).

*F ADC* and *OS*. A similar pattern was observed for F ADC in Scan1, with median OS 43 ± 7.3 weeks for *F ADC* ≥ 1 (n = 6) and median OS 26 ± 5.2 weeks for *F ADC* < 1 (n = 7) (p = 0.15, Fig. 2c) and in Scan 2 with median OS 65 ± 24.1 weeks (n = 5), and 26 ± 4.2 weeks (n = 8) (p = 0.014, Fig. 2d).

*F f, F SUV*_max_ and *OS*. Similar analyses for *F f* and *F SUV*_*max*_ showed no difference in OS for Ff and F*SUV*_*max*_ ≥ 1 and < 1 (*F f*: p = 0.582 at week 5, p = 0.416 at week 9; *F SUV*_*max*_: p = 0.755 at week 5, p = 0.867 at week 9).

Regarding the non-injected no differences or correlations between groups were observed.

### Correlations between changes of DWI/PET parameters and OS

**Figure 3:**
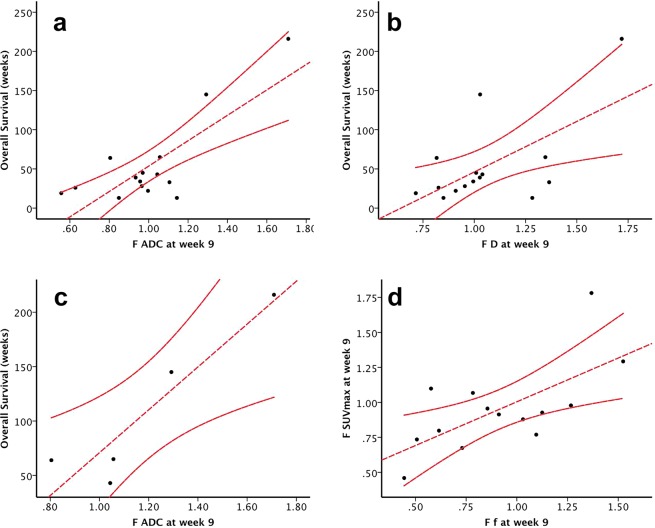
Correlation analysis of overall survival (OS) with D, and subsequently ADC, in the injected metastases. The Pearson’s correlation analysis was used. (**a**) *F D* correlation with OS at week 9 (Scan 2) including all patients (n = 21, p = 0.015, R^2^ = 0.378). (**b**) *F ADC* correlation with OS at week 9 (Scan 2), including all patients (n = 21, p < 0.01, R^2^ = 0.628). (**c**) Correlating OS in the group of patients that were “responders” in our previous publications^36,51^ based on PET/CT evaluations (n = 6) with fold change (*F*) *F ADC* in Scan 2 at week 9 (p = 0.023, R^2^ = 0.861). (**d**) *F f* values in the injected metastases were correlated to *F SUV*_*max*_ values at week 9 (Scan 2), including all evaluable patients (n = 21, p = 0.01, R^2^ = 0.442). The Pearson’s correlation analysis was used.

Increase of ADC and D values in the injected metastases in Scan2 correlated to OS. *F D* and F *ADC* correlated to OS in Scan2 (p = 0.015, R^2^ = 0.378, p < 0.01, R^2^ = 0.628, respectively; Figs. 3a,b).

In the “responders” patients (based on FDG-PET/CT: #2, #4, #7, #8, #14, #18)^36,38^, the *F ADC* in Scan2 was positively correlated with OS (p = 0.023, R^2^ = 0.861, Fig. 3c). We performed the same analyses for SUV_*max*_ and no correlation was found with OS (p = 0.946 at week 5, p = 0.718 at week 9).

Regarding the non-injected metastases, no differences or correlations between groups were observed.

### Correlation between FDG-PET/CT and DW-MRI in the injected lesion

*F f* values and *F SUV*_*max*_ in Scan2 were positively correlated (p = 0.01, R^2^ = 0.44; Fig. 3d) while correlations between the rest of DWI parameters and SUV_*max*_ were poor with values of R^2^ not exceeding 0,3 (Supplemental Material, Fig. 5s–7s).

The same analyses for SD and PD groups as well as for PMR, PMD and SMD groups showed no relationship between *SUV*_*max*_ and was similar (p = 0.765 at week 5, p = 0.620 at week 9).

### Relationship between OS and change in DWI/PET parameters

#### All evaluable patients (n = 21)

The hazard ratios, 95% confidence intervals (CI) and p-values for univariate Cox regression analyses of DWI and PET parameters at week 5 and 9 are presented in Table 3. There were two significant results: for F *ADC* in Scan2 (HR 0.02, p = 0.013) and F *D* in Scan1 (HR = 0.004, p = 0.007). Both results showed that an increase in these parameters were related to improved survival. A similar strong trend was observed for F *ADC* in Scan1 (HR 0.04, p = 0.052) as well as for F *D* in Scan2 (HR = 0.08, p = 0.063).

**Table 3:** All evaluable patients (n = 21).

Variable	HR	95% CI	P-value
F *SUVmax* w5	0.92	(0.16–5.20)	0.923
F *SUVmax* w9	1.92	(0.35–10.70)	0.455
F *ADC* w5	0.04	(0.00–1.02)	0.052
F *ADC* w9	0.02	(0.00–0.44)	0.013
F *D* w5	0.004	(0.00–0.22)	0.007
F *D* w9	0.08	(0.01–1.15)	0.063
F *f* w5	2.90	(0.55–15.30)	0.209
F *f* w9	0.58	(0.11–3.02)	0.519

The hazard ratios, 95% confidence intervals (CI) and p-values for univariate Cox regression analyses of DWI and PET variables at week 5 (Scan 1) and 9 (Scan 2) are presented in Table 3. There were two significant results: for F *ADC* in Scan 2 and F *D* in Scan 1. Both results showed that an increase in these parameters was related to improved survival. A similar strong trend was observed for F *ADC* in Scan1 and for F *D* in Scan2.

**Figure 4:**
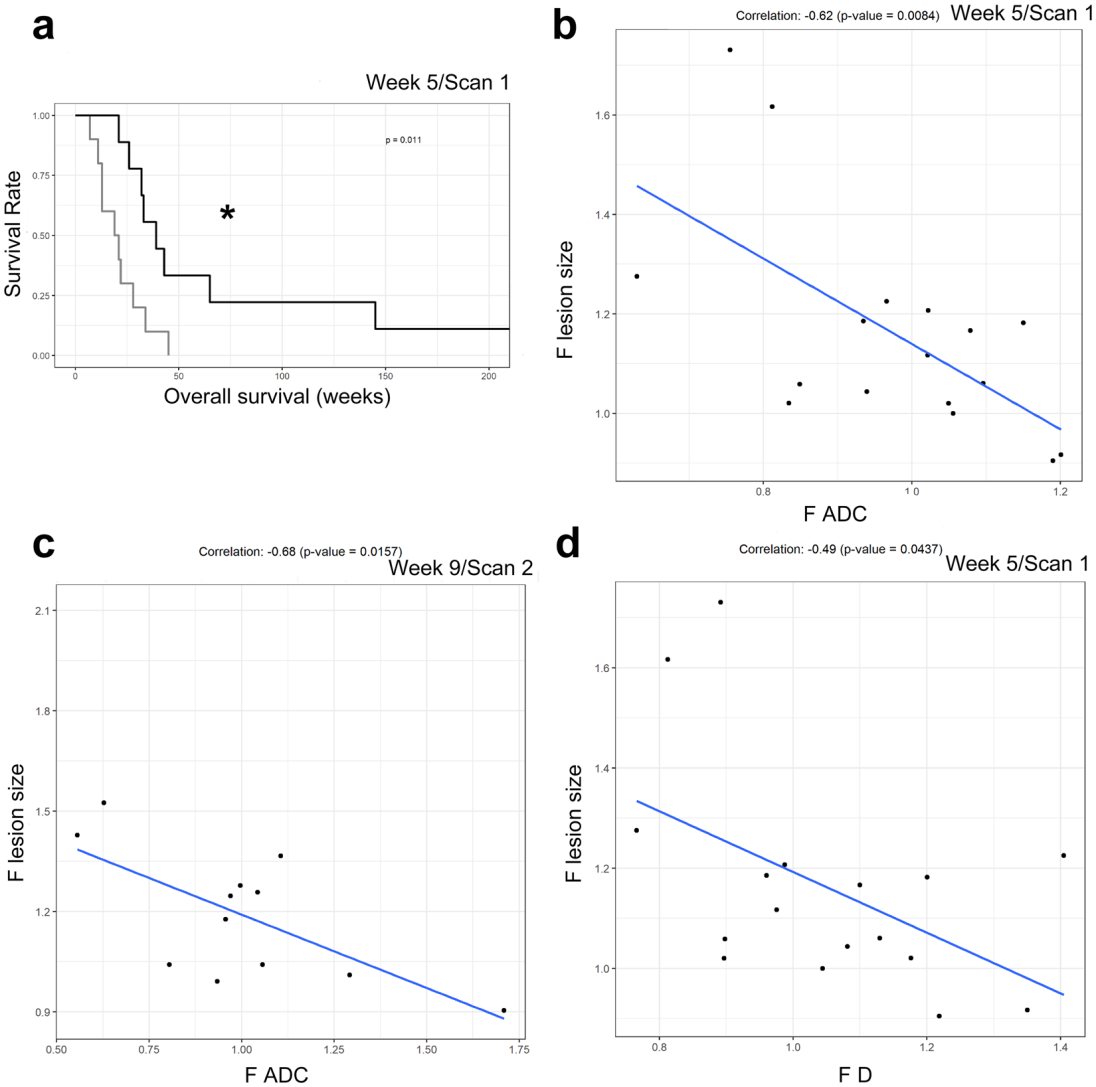
(**a**) Patients with F *D* value above the median had a significantly better OS at week 5 (Scan 1), including all patients (n = 21, p = 0.011). The black line shows the F *D* value above the median, while the grey line shows F *D* value below the median.(**b**) Correlation for F *ADC* with the fold change of the lesion size based on RECIST 1.1 at week 5 (Scan 1). The gray line is the fitted regression line. The correlation coefficient was −0.62 and p = 0.008. (**c**) Correlation for F *ADC* with the fold change of the lesion size based on RECIST 1.1 at week 9 (Scan 2). The gray line is the fitted regression line. The correlation coefficient was −0.68 and p = 0.016. (**d**) Correlation for F *D* with the fold change of the lesion size based on RECIST 1.1 at week 5 (Scan 1). The gray line is the fitted regression line. The correlation coefficient was −0.49 and p = 0.044. (* indicates p < 0.05).

In order to evaluate our methodology, the test for relationship between OS and DWI/PET parameters was repeated but the parameters were categorized by their median value. In Scan1, at week 5, patients with F *D* value above the median had a significantly better OS (p = 0.011; Fig. 4a). A similar trend was observed in Scan2, at week 9 (p = 0.065; Supplemental Material, Fig. 8s).

### Relationship between change in lesion size (CT) and change of DWI/PET parameters

The mean size of the injected lesions was 50 mm (11 mm −114 mm).

The relationship between changes in lesion size (CT) and changes in ADC as illustrated in Fig. 4 (b,c) was, with significance for both post treatment scans (correlation coefficients: −0.62, p = 0.0084 at week 5; − 0.68, p = 0.0157 at week 9). The relation is negative where an increase in ADC is related to a decrease in lesion size. For D a similar relationship with lesion size in Scan1 is shown in Fig. 4d (−0.49, p = 0.0437). In Scan2 a similar trend was observed (−0.51, p = 0.0914; suppl).

Changes in lesion size were not related to f or SUV_*max*_. Increase ≥ 30% of the size of the injected metastasis in PET/CT and MRI Scan2, was associated with shorter OS [p = 0.001, median OS for < 30%: 45 ± 3 weeks (n = 9), for ≥30% 19 ± 7.5 weeks (n = 4), Supplemental Material, Fig. 9s].

### Comparisons between fold changes of DWI/PET parameters and reference response criteria

#### RECIST 1.1 in CT and MRI

All evaluable patients (n = 21): The treatment resulted in stable disease (SD) in 11 patients and PD in 10 according to Scan1 at week 5. In Scan2, at week 9, there were 8 patients with SD and 8 with PD.

Patients who underwent all the examinations (n = 13): SD was achieved in 9 and PD in 4 patients Scan 1. In Scan 2, 8 patients were assessed as having SD and 5 as PD.

*F ADC, F D, F f* and *F SUV*_*max*_ in the injected and not injected metastases were similar in SD and PD groups as was the OS.

#### EORTC criteria in FDG-PET/CT

All evaluable patients (n = 21): There were 12 patients who achieved stable metabolic disease (SMD), 4 had partial metabolic response (PMR) and 5 had progressive metabolic disease (PMD). Four out of five PMD patients had developed new lesions in Scan 1. In Scan 2, 8 patients had SMD, 2 PMR and 6 PMD. Five out of the 6 PMD patients had developed new lesions.

**Figure 5:**
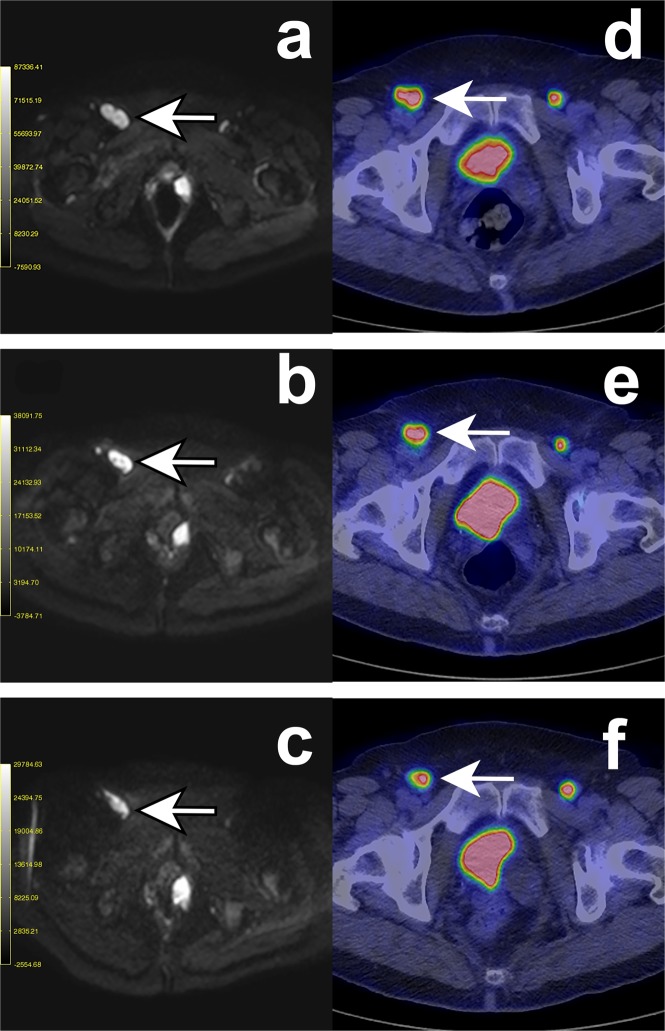
DW- and PET-images of a 67-year-old woman with disseminated mucosal melanoma in vulva (cohort 2). Four intratumoral injections with AdCD40L were given to a right inguinal lymph node metastasis. The patient had partial metabolic response (PMR) in the first and stable metabolic disease (SMD) in the second post-therapy evaluation, according to EORTC criteria while the case was assessed as stable disease (SD) at both post-therapy scans according to RECIST 1.1. An increase in ADC/D in the injected metastasis was observed at both DW-MRI scans post-therapy. The short axis of the injected metastasis was measured at each time date and was unaltered according to the RECIST 1.1 criteria. An initial decrease of the f% value was observed while it was increased at the second DW-MRI scan post-therapy. In a not injected left inguinal lymph node metastasis the ADC-value was increased at both DW-MRI scans post therapy while the value of D was initially increased but it was decreased in the second post-therapy evaluation. A similar pattern as for the D-value was observed regarding the values of f% while the size of the metastasis was unaltered. A third metastasis at the proximity of the urinary bladder is present in the DW-images. This metastasis was not distinguishable at the PET/CT scans due to the high activity of the urinary bladder. **(a–c**) Diffusion-weighted MR image (DWI) at b = 900 s/mm^2^ -in the axial plane. The arrows indicate the injected right inguinal metastasis at baseline (**a**), in Scan 1 at week 5 (**b**) and in Scan 2 at week 9 (**c**). (**d–f**) FDG-PET images. The arrows indicate the injected right inguinal metastasis at baseline (**a**), in Scan 1 at week 5 (**b**) and in Scan 2 at week 9 (**c**).

Five patients with a positive therapy response, *i.e*. PMR on FDG-PET/CT showed an increase in D/ADC. One of these patients is presented in Fig. 5. The ADC maps are presented in the Supplemental Material section (Supplemental Material, Fig. 10s).

When only patients undergoing all the examinations (n = 13) were included in the analysis, there were 8 patients with SMD, 2 with PMR and 3 with PMD in Scan 1. In Scan 2, 8 had SMD, 2 PMR and 3 PMD.

*F ADC, F D, F f* and *F SUV*_*max*_ in the injected and not injected metastases were similar in the SMD, PMD and PMR groups as was the OS. All the new lesions detected by FDG-PET/CT were also detected by the DW-MRI.

### Change in DWI and PET parameters

The DWI variables were similar at baseline, Scans 1 and 2 (Wilcoxon signed-rank test). Furthermore, a multivariate permutation test was performed on the measurements, still not showing any significant differences in the DWI parameters between baseline and follow-up Scan 1 and 2 (Table 4). Also, the SUV_*max*_ was similar at baseline and follow-up PET/CT 1 and 2 (Wilcoxon signed-rank test).

**Table 4:** A multivariate permutation test (MPT), a combined test of all three univariate tests, was performed on the DWI measurements.

Time	ADC		D		f		Combined
	Median (IQR)	P-value	Median (IQR)	P-value	Median (IQR)	P-value	P-value (MPT)
w5	1.02 (0.23)	0.5412	1.03 (0.26)	0.4900	0.84 (0.39)	0.1232	0.3324
w9	0.97 (0.19)	0.8040	1.01 (0.28)	0.7197	0.86 (0.51)	0.2293	0.6039

The null hypothesis was that all three null hypotheses for the univariate tests hold and the alternative hypothesis was that at least one of the null hypotheses was not true. PMT showed no significant differences in the DWI parameters between baseline and follow-up Scan 1 and 2.

## Discussion

Our results show the potential of MRI measuring ADC and IVIM-parameters to assess tumor response to immunotherapies as exemplified in this study on local immunomodulatory AdCD40L-gene therapy in patients with refractory metastatic MM. To our knowledge, this is the first report on DW-MRI for tumor response evaluation in MM patients treated with immunomodulation. Thus, based on DWI parameters, D and ADC, we were able to identify a subgroup of patients with significantly longer median OS when *D* and/or ADC were increased. Based on D we were already in the first evaluation at week 5 able to identify a subgroup of patients with significantly longer median OS when F *D* ≥ 1. Regarding ADC changes at week 9 enabled the identification of a subgroup of patients with improved survival.

An increase in SUV_max_ during treatment can either be related to an inflammatory therapy response or to tumor progression [26]. When tumor necrosis occurs, the decreased cellularity results in higher D/ADC values. These two circumstances could explain that D/ADC, but not SUV_max_, was significantly positively correlated to OS. This is also in line with our finding that at least five patients who experienced clinical benefit showed positive therapy response (PMR), with a concomitant increase in D/ADC. One confounding parameter is the melanin content of the tumor, which can cause susceptibility effects affecting the quantitative parameters of DWI^39^. This potential confounder was to some extent accounted for, by evaluating the degree of curve fitness for the mono- and bi-exponential fit.

There are previous reports on DW-MRI to assess early response to chemotherapy, but not to immunotherapy^40–42^. Interestingly, the increase in ADC, reflecting therapy-induced apoptosis or necrosis^27,29,43,44^, has been reported to precede size reduction and may thus potentially be used to early detect response to immunotherapy^45^. This is in line with our results in this imaging sub-study where the change in size of the injected metastasis was negatively correlated to change in *ADC* in both post-treatment scans while for D it was only apparent at the first post-treatment scan.

Notably, the finding that OS correlated to the increase of D and ADC in the injected metastases, from baseline to Scan1 and Scan2 respectively but not to SUVmax changes during the same time interval, clearly suggests that DW-MRI might be superior to FDG-PET/CT for early prediction of response in the present setting. The reasons that FDG-PET was not helpful for therapy monitoring are unclear. The recently published report from the European Association of Nuclear Medicine (EANM) 2017 Annual Congress recommends that the first immunotherapy response evaluation should be performed at 8 or 9 weeks after initiation of immunotherapy^35^. Our first FDG-PET/CT follow-up at week 5 from treatment start may therefore have been performed too early, whereas the time point for Scan2 at week 9 are in line with these recommendations. Another contributing factor may be a remaining inflammatory response in the tumors which counteracted the therapy induced decrease in SUV_max_ because of apoptosis. Further, a decrease in the SUV_max_ may only reflect a metabolic effect on the tumor cells by the therapy that does not result in apoptosis. These effects were however not evident in the DWI measurements. Despite that fact that an inflammatory response would increase the cellularity, and result in a D decrease, such an effect will be counteracted both by the inflammatory edema, the therapy-induced deterioration of the cell membrane functions and apoptosis, which altogether will increase D.

The positive correlation between *F f* and *F SUV*_*max*_ indicates that f increases in parallel to the tumors´ metabolic activity possibly reflecting a more organized capillary network supporting the tumor.

In the relationship between OS and changes in DW-MRI parameters it is unclear to what extent they reflect, on one hand, the treatment effect and, on the other hand, the tumor´s aggressiveness. Because FDG-PET/CT and DW-MRI reflect different aspects of tumor biology related to therapy effects, the combined use of these modalities might have the best potential to assess early therapy effects.

Our PET response assessment relied on the EORCT criteria, which in recent studies have been shown less satisfactory for immunotherapy response evaluation in metastatic melanoma patients. However, since we performed the evaluation of the first study cohorts before the publication of these findings and the introduction of iPERCIST, we have for the sake of consistency throughout the study chosen to continue applying the EORTC criteria.

One could argue that the addition of cyclophosphamide and/or radiotherapy might have an impact on the functional imaging parameters. Conditioning by cyclophosphamide is used in clinical trials in order to enhance the efficacy of immunotherapy^46^. In cohort II, the patients were conditioned with such a low dose of cyclophosphamide that no cytotoxic effect against melanoma can be expected. Radiotherapy can enhance antitumor immune responses in several ways^47^. Melanoma has historically been considered a radioresistant tumor^48^. In cohort III, the patients were treated with the same protocol as in cohort II with the addition of a single-fraction of radiotherapy in order to facilitate antitumor immune responses. Therefore, the rational of conditioning by cyclophosphamide and/or adding radiotherapy was to enhance the efficacy of immunotherapy. The statistical analysis of the DWI and PET parameters in the injected metastases showed that they all were similar in the three groups. Consequently, neither cyclophosphamide nor radiation therapy did not seem to have an impact on the functional imaging parameters in the present sub-study.

The present study has a few major limitations. Firstly, the patients harbored different types of MM, with the risk of introducing heterogeneity regarding tumor biology. Secondly, the limited number of patients as well as the factum that there were three different cohorts. Only thirteen patients completed all imaging time points designated in the trial. Nevertheless, as we argued above neither the addition of cyclophosphamide (cohort 2) nor the 8 Gy fraction of radiotherapy (cohort 3) in our imaging sub-study seems to have influenced the imaging parameters. Merely thirteen patients completed imaging on all time points during the trial and the therapy effects were not evaluated for all metastases, because of the restricted field of view of the present MRI examination protocol. We have adopted SUVmax in order to quantify the tumor FDG-uptake since it constitutes a user independent, reproducible measurement value when applying ROIs or VOIs. According to the repeatability issue, the intratumoral injections of our 24 patients were administered in metastases in different organs thereby making it impossible to harmonize the ADC, IVIM-parameters according to the recommendations by QIBA^34^. Therefore, we decided to define the fold change of relevant functional imaging parameters as a fraction of the post-treatment/pre-treatment measurement. We did not find any significant changes caused by the treatment, in the non-injected lesions, that could support a systemic effect prolonging OS.

This is a small cohort in a phase I/IIa study where the primary endpoint was the efficacy of the investigational novel drug AdCD40L. One of the secondary exploratory endpoints was indeed tumor response using functional imaging techniques to investigate the potential value of quantitative imaging as markers for response/prognosis. The study protocol was written in 2010 when neither FDG-PET/CT either DW-MRI had an obvious role in assessing tumor response to immunotherapy and the imaging time-points were rather of an exploratory type.

Our results need to be further validated in a larger patient population with similar type of MM, using a more detailed whole-body approach also for DW-MRI. The latter would, however, considerably increase the examination time, probably in an unacceptable way in the daily clinical routine. Nevertheless, *F ADC* ≥ 1 and *F D* ≥ 1 were both associated with higher median OS. An increase from baseline to post-treatment of ADC and D correlated significantly with OS.

In conclusion, our results indicate that DW-MRI is useful to identify early responders to local immunostimulatory AdCD40L gene therapy. Further studies are needed to verify these findings and to assess whether DW-MRI represent a useful method for early evaluation of response to immunotherapy in general.

## Materials and Methods

### Trial design

The phase I/IIa study (NCT01455259, registration date 19/10/2011; Sponsor’s Protocol Code Number, 002:CD40L) was conducted in compliance with our protocol (EudraCT 2010-023103-94, date of registration 26/09/2010), in accordance with the international conference of harmonization – good clinical practice guidelines, the principles of the Declaration of Helsinki and applicable regulatory guidelines, and was approved by the Regional Ethical Review Board in Uppsala and the Medical Products Agency. The patient characteristics, feasibility, safety and efficacy of the AdCD40L-treatment have been published previously^36,37^. Briefly, 24 patients (mean age 62.5 years, 23–79 years) with refractory disseminated, histologically proven, MM with ≥ 2 measurable lesions according to the RECIST 1.1, were enrolled October 2011-January 2015. Specifically, 11 men (mean age 61, 23–79 years) and 13 women (mean age 64, 45–72 years) were included. Signed informed consent was obtained from all subjects. Exclusion criteria were: pregnancy, other malignancy, life expectancy < 3 months, significant illness and severe systemic autoimmune disease. The majority of the patients had ocular melanoma. All the patients received all established treatments. One third of the patients received some type of immunotherapy prior to study enrollment^37^. All other therapies were, however, stopped due to PD before the 10-week DWI- and PET- monitoring period. In fact, only 2 out of 24 patients (patient #8 and #9) received other treatments after AdCD40L. Four years after the last AdCD40L-injection patient #8 had received nivolumab for 2 months. Patient #9 was treated shortly with BRAF-inhibitor^49^. In cohort I, 6 patients received four weekly ultrasound-guided intratumoral injections of 2.5 × 10^11^ virus particles AdCD40L [AdCD40L vector cloned at the CAGT at Baylor College of Medicine, Houston, TX]. In cohort II, 9 patients were conditioned with low-dose intravenous cyclophosphamide (300 mg/m^2^) 1–2 days prior to the first and fourth injection. In cohort III, 9 patients were treated with the same protocol as in cohort II with the addition of a single-fraction of radiotherapy one week prior to the first AdCD40L injection. The injected metastasis was chosen based on its accessibility for a safe intratumoral injection. The four weekly injections and the single fraction of radiotherapy were delivered in the same selected lesion. The first injection was given at week 0. Regarding the non-injected metastasis, we selected the one with the highest SUV max uptake that at the same time was in the DW-MRI field of view.

The locations of the injected metastases were the following: 13 in the liver, 7 in subcutis, 3 in lymph nodes and one in the parotid gland.

The locations of the non-injected metastases: 8 in lymph nodes, 4 in the liver, 2 in the lungs, 2 in subcutis, 2 in bone and one in the adrenal gland. In 3 patients the non-injected metastases with the highest SUVmax uptake were not within the field of view of the DW-MRI scans.

In this imaging sub-study of the phase I/IIa study with AdCD40L, FDG-PET/CT and DW-MRI were performed pretreatment -one to two weeks before treatment initiation- and repeated after two and six weeks after the last injection, resulting in the first evaluation being scheduled at week 5 (Scan1) and the second evaluation at week 9 (Scan2).

All patients (n = 24) have been previously reported^36,37^. These two prior reports comprised the radiological evaluation of the study subjects by FDG-PET/CT and WB-MRI according to RECIST 1.1 as well as by FDG-PET in the injected metastases according to EORTC criteria. In the present manuscript we report for the first time our DW-MRI findings in the injected and in one non-injected metastasis per patient. Further, we report FDG-PET data of the non-injected metastases according to EORTC criteria.

#### FDG-PET/CT

Whole body FDG-PET/CT was performed on a Discovery ST16 PET/CT scanner (GE Healthcare, Little Chalfont, Great Britain). The patients underwent a low-dose non-contrast-enhanced CT scan (120 kV, auto mA 20–100 mA, noise index 28, Pitch 1,375:1) with 3,75 mm slice thickness, matching that of the PET emission. After 4 hours of fasting the patients received an intravenous bolus injection of mean 185 MBq FDG (2 MBq/kg). Image acquisition began one hour after injection. A CT scan for attenuation correction was followed by PET emission, 3 minutes per bed position, from head to toe. Attenuation-corrected emission images were reconstructed with an iterative algorithm and the reconstructed images were recalculated to standardized uptake value (SUV) images, corrected for patient body weight and amount of injected FDG. The software that was used for ROI definition and SUVmax read out in this study was Carestream Vue PACS version 11.3.1.1 (Carestream Health, NY, USA). The tumors were delineated with circular regions of interest (ROIs) in the PET section measuring the SUV_max_^50^. The assessment of the PET examinations was performed by professor AS who has 27 years of experience. All measurements of SUVmax in both injected and not injected metastases were repeated three times and the results were similar.

#### DW-MRI

MRI utilized a 1.5 Tesla clinical Philips Achieva system (Best, The Netherlands). The morphological T1 and T2 sequences covered from top of the head to upper thighs. The slice thickness was 8 mm and 0.8 mm gap, 25 slices. Respiratory-triggered sequence TE therefore varied but was set to minimum of 63–72 ms. At the level of the injected lesion, a single shot (SS) - echo planar imaging (EPI) sequence with DW-MRI technique was used including 7 b factors (0, 50, 100, 150, 300, 600, 900 s/mm^2^) and an isotropic diffusion-sensitizing gradient scheme. ADC maps and maps of the D were automatically calculated by using a mono-exponential and a bi-exponential fit, respectively, to all b-factors (Philips DWI_Tool R1.5). The degree of curve fitness was checked for both the mono- and biexponential fit at each measurement. The ROIs in the DW images were drawn in lesions larger than 1 cm including as much as possible of the lesions but without risking the influence of partial volume effects.

ROIs were drawn to include the injected and one non-injected lesion in the same field of view of the respective parametric maps, mean ADC, mean D to allow for extraction of the f values ± standard deviations at each examination enabling longitudinal monitoring of the lesions. The assessment of the MRI examinations was performed by professor HA who has 31 years of imaging experience.

#### Data analysis

*Reference response criteria*. Morphological response^14^ was defined according to RECIST 1.1[9] using the low-dose non-contrast-enhanced CT scan when the injected metastases were subcutaneous metastases or localized in lymph nodes. In the case of liver metastases, a double control with the T2-weighted MR sequence was performed to ensure the accuracy of our measurements. Metabolic response was defined according to EORTC criteria^22^.

*Statistical data analyses*. The statistical analyses were performed using SPSS 20.0 (IBM, USA) and R Statistics. The data were evaluated by a group of professional statisticians (Statisticon; Statistics & Research, Uppsala, Sweden). In each patient two metastases were selected: the injected tumor and the lesion with the highest SUV_max_ that also was measurable on MRI (≥1 cm) and located within the DW-MRI field of view. The changes in ADC, D, f, SUV_max_, and size of the lesions were calculated as fractions (fold changes = F): e.g. *F ADC* = ADC_post_/ADC_pre_. “Pre” represents values obtained pretreatment and post values were obtained at week 5 (Scan1) or 9 (Scan2). This relative change (week 5 or 9 value/baseline value) assumes that a change from a low starting value is larger than an equal change from a higher starting value. For the relative change 1 (F-value = 1) indicates no change from baseline. *F*-value ≥ 1 indicates increase while < 1 decrease of the chosen parameter. The Kaplan-Meier method followed by log-rank test used to compare the OS in patients with F ADC, F D, F f, F SUV_max_ and F of lesion size ≥ 1 and < 1. The same method was used to compare the OS with the median value of every parameter.

Pearsson´s correlation analysis was performed for both OS and *F ADC, F D, F f, F SUV*_*max*_ and size. Correlations between FDG-PET and DW-MRI parameters employed Pearsson´s correlation analysis. Univariate Cox proportional hazard regression analysis for the DWI and PET variables at week 5 and 9 was used for the relationship between OS and those parameters. Wilcoxon signed-rank test and a multivariate permutation test, which is a combined test of all three univariate tests were used for the DWI variables and the SUVmax.

Differences between the three cohorts, RECIST 1.1 and EORTC criteria regarding *F ADC, F D, F f, F SUV*_*max*_ and OS were investigated with one-way ANOVA followed by planned contrasts when data were normally distributed. Kruskal-Wallis followed by Mann-Whitney *U*-tests were performed when data were not normally distributed. Paired t-tests were used for direct comparisons between the SUV_*max*_ pre, at week 5 and 9. A p ≤ 0.05 was considered significant.

## Supplementary information

Supplementary Info Schiza et al

## Data Availability

All the data including PET and DW-MRI assessments and statistical analyses are available upon reasonable request.
