# Supplementary material for: Evaluation of Diffusion-Weighted MRI and FDG-PET/CT to Assess Response to AdCD40L treatment in Metastatic Melanoma Patients
Source: Sci Rep. 2019 Dec 2;9:18069. doi: 10.1038/s41598-019-54438-x (PMC6889008; doi:10.1038/s41598-019-54438-x)
Supplement: Supplementary file 1 — Supplementary Info Schiza et al [file 41598_2019_54438_MOESM1_ESM.pdf]

Evaluation of Diffusion-Weighted MRI and FDG-PET/CT to Assess Response to AdCD40L treatment in Metastatic Melanoma Patients

Aglaia Schiza[1,2], Sandra Irenaeus[1,2], Francisco Ortiz-Nieto[3], Angelica Loskog[1], Thomas Tötterman[1], Anders Sundin[3,4], Gustav J. Ullenhag [1,2*], Håkan Ahlström[3,4,5*]

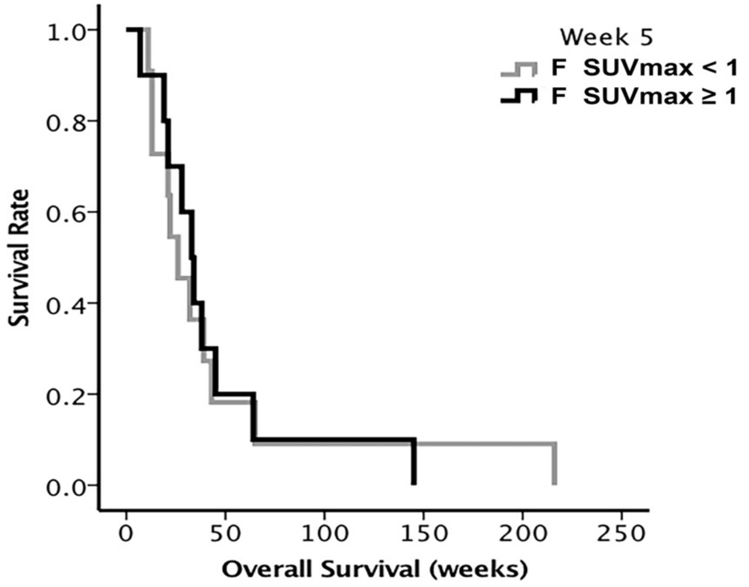

Figure 1s

Median OS between patients with F SUV_{max} ≥1 (black lines) and compared with those with F SUV_{max} <1 (grey lines) at week 5 (p=0.968).

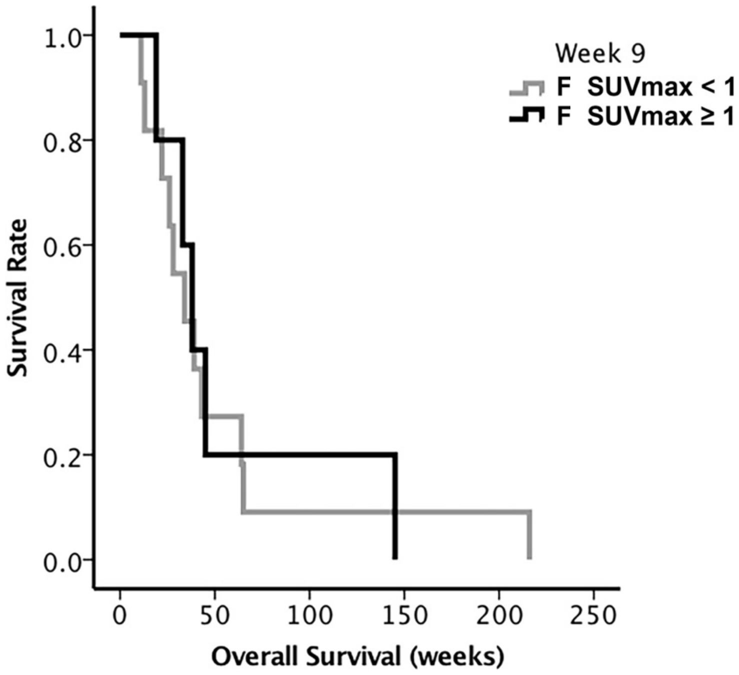

Figure 2s

Median OS between patients between patients with F SUV_{max} ≥1 (black lines) and compared with those with F SUV_{max} <1 (grey lines) at week 9 (p=0.905).

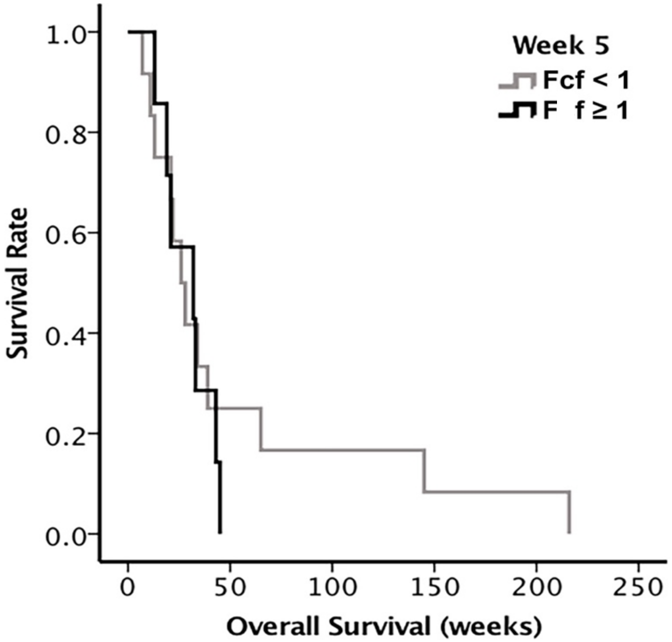

Figure 3s

Median OS between patients between patients with F *f* ≥1 (black lines) and compared with those with F *f* <1 (grey lines) at week 5 ($p=0.556$).

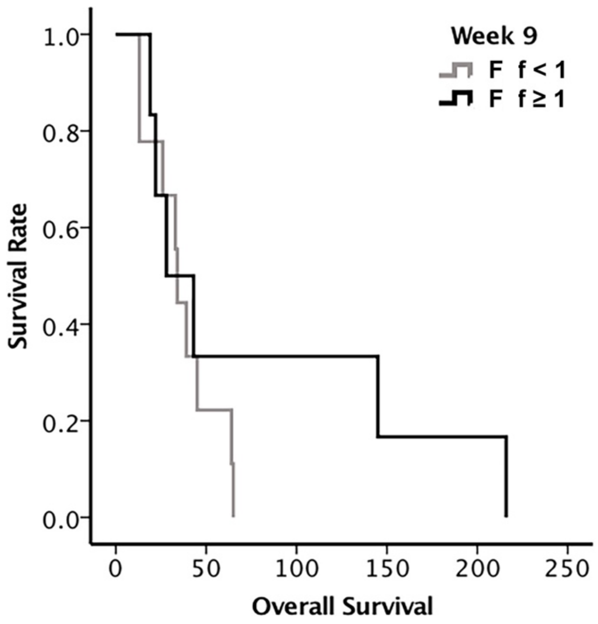

Figure 4s

Median OS between patients between patients with F $f \geq 1$ (black lines) and compared with those with F $f < 1$ (grey lines) at week 9 ($p=0.348$).

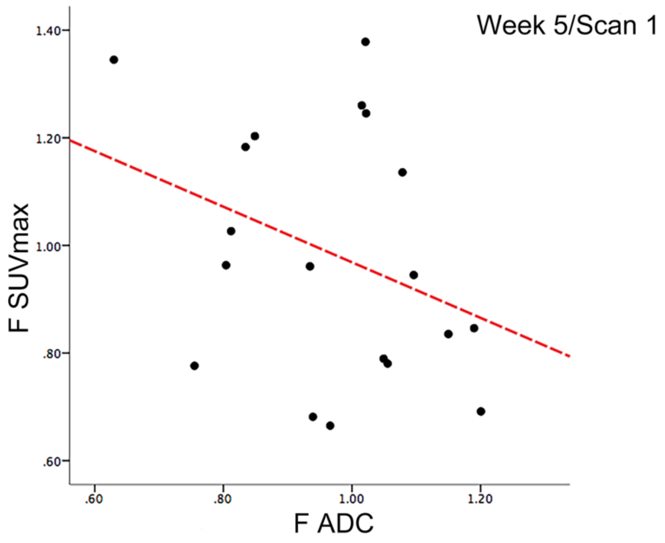

Figure 5s

F ACD values were correlated to *F* SUV_{max} values at week 5 (Scan 1) in the injected metastasis including all evaluable patients in the analysis (n=21, p=0.015, R^2=0.117). The Pearson's correlation analysis was used.

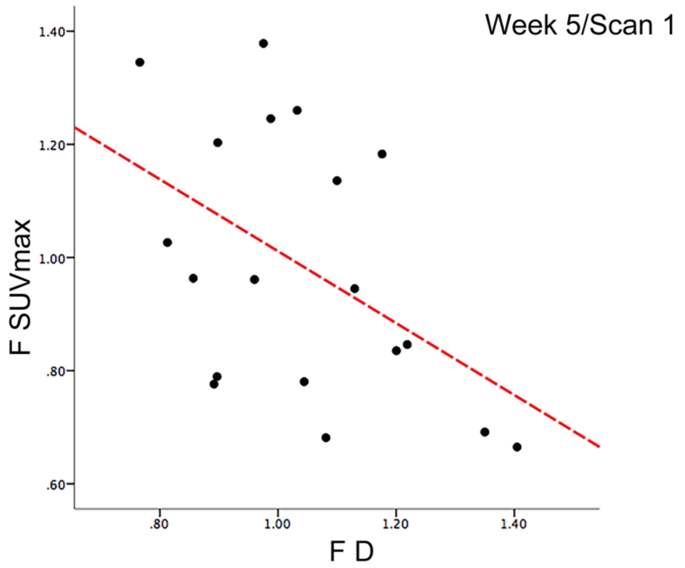

Figure 6s

F D values were correlated to *F* SUV_{max} values at week 5 (Scan 1) in the injected metastases, including all evaluable patients in the analysis (n=21, p=0.039, R^2=0.227). The Pearson's correlation analysis was used.

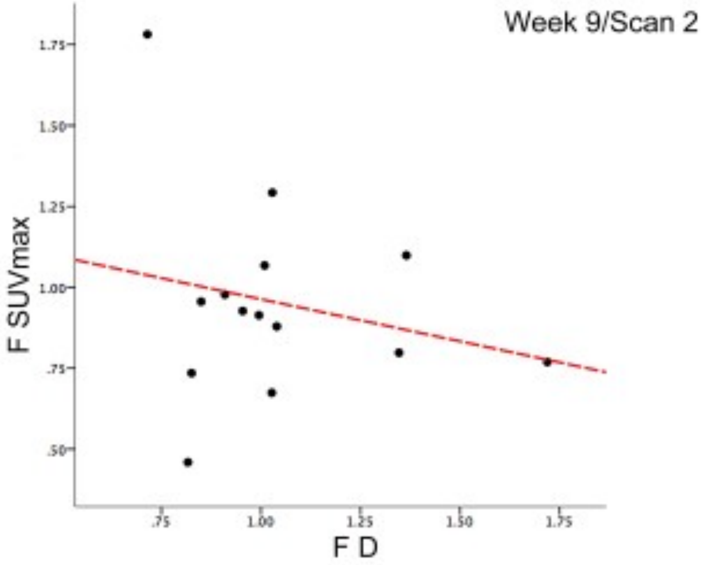

Figure 7s

F D values were correlated to *F* SUV_{max} values at week 9 (Scan 2) in the injected metastases, including all evaluable patients in the analysis (n=21, p=0.445, R^2=0.049). The Pearson's correlation analysis was used.

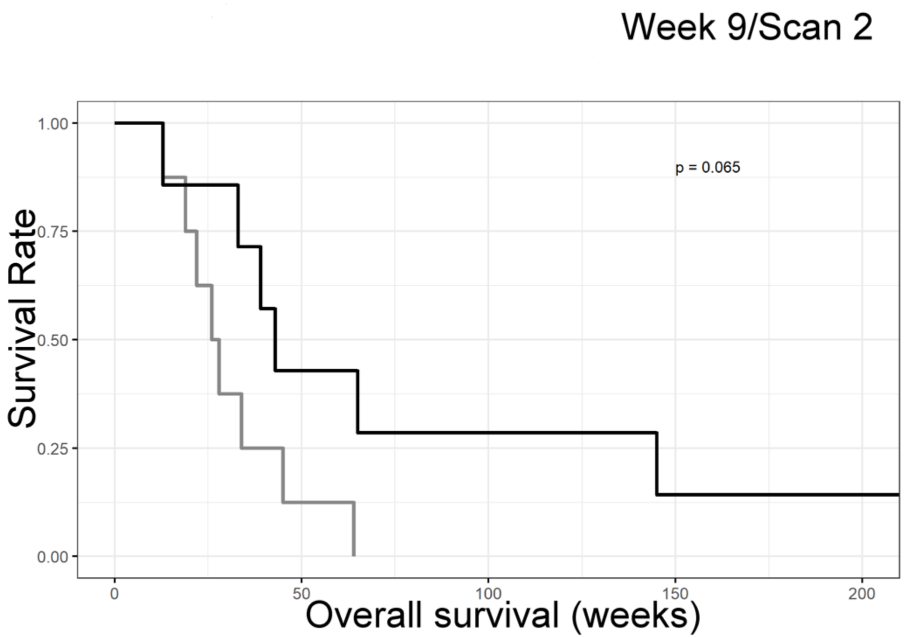

Figure 8s

Patients with F *D* value above the median had a better OS at week 9 (Scan 2), including all patients (n=21, p=0.065). The black line shows the F *D* value above the median, while the grey line shows F *D* value below the median.

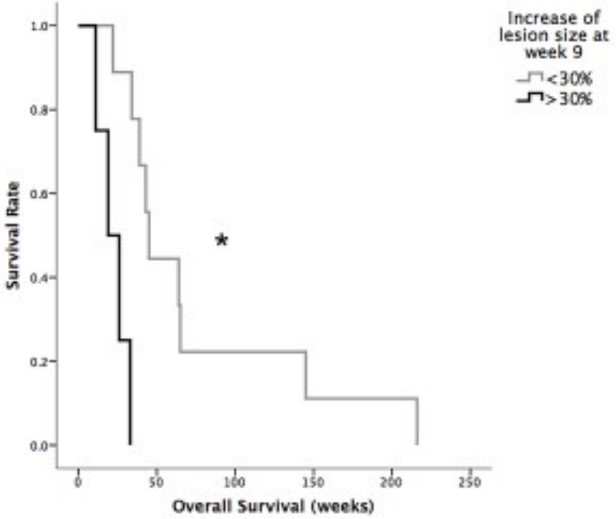

Figure 9s

Comparing OS in the group of patients that had an increase ≥30% of the size of the injected metastasis at week 9 (Scan 2) with the group with increase < 30% (p=0.001). The Kaplan-Meier method was used followed by log-rank test (* indicates p <0.05).

Figure 10s

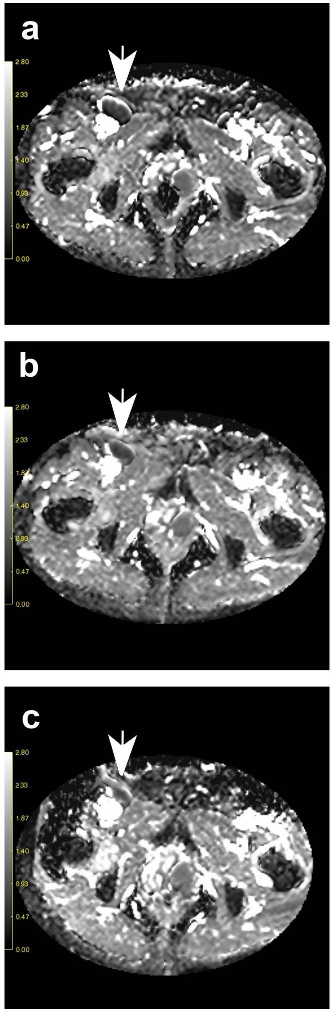

ADC maps of a 67-year-old woman with disseminated mucosal melanomainvulva(cohort2).InjectionswithAdCD40L weregiventoa right inguinal lymph node metastasis. The patient had partial metabolic response(PMR)inthefirstandstable metabolicdisease(SMD)inthe second post-therapy evaluation, according to EORTC criteria while the case was assessed as stabledisease(SD)atbothpost-therapyscans according to RECIST 1.1. An increase in ADC/D in the injected metastasis wasobservedatbothDW-MRIscanspost-therapy.The longest diameter of the injected metastasis was measured at each time dateandwasunalteredaccordingtotheRECIST1.1criteria.Aninitial decreaseofthef%valuewasobservedwhileitwas increasedatthe second DW-MRI scan post-therapy. In a not injected left inguinal lymph node metastasis the ADC-value was increased at both DW-MRI scans post therapy while the value of D was initially increased but it was decreasedinthe secondpost-therapyevaluation.Asimilarpatternas fortheD-valuewasobservedregardingthevaluesoff%whilethesize ofthe metastasiswasunaltered.Athirdmetastasisattheproximityof theurinarybladderispresentintheimages.Thismetastasiswas not distinguishable at the PET/CT scans due to the high activity of the urinary bladder.

a-c: ADC maps. The arrows indicate the injected right inguinal

metastasis at baseline (a), at week 5 (b) and at week 9 (c).

Table 1s:

Kruskal-Wallis test was performed on the DWI, PET and lesion size measurements. The null hypothesis was that the distribution of fold changes of DWI, PET and lesion size parameters is the same across the three cohorts. The test showed no significant differences in the fold changes of DWI, SUVmax and lesion size between baseline and follow-up Scan 1 and 2.

	**F ADC**	**F D**	**F f**	**F SUVmax**	**F lesion size**
**Time**	**p-value**	**p-value**	**p-value**	**p-value**	**p-value**
w 5 (Scan 1)	0.30	0.23	0.46	0.87	0.95
w 9 (Scan 2)	0.22	0.91	0.48	0.17	0.16
